# Continuous Goos-Hänchen Shift of Vortex Beam via Symmetric Metal-Cladding Waveguide

**DOI:** 10.3390/ma15124267

**Published:** 2022-06-16

**Authors:** Xue Fen Kan, Zhi Xin Zou, Cheng Yin, Hui Ping Xu, Xian Ping Wang, Qing Bang Han, Zhuang Qi Cao

**Affiliations:** 1 College of Internet of Things Engineering, Hohai University, Changzhou 213022, China; kanxf.tt@foxmail.com (X.F.K.); hqb0092@163.com (Q.B.H.); 2 Changsha Lubang Photoelectric Technology Co., Ltd., Changsha 410023, China; zousilong@foxmail.com; 3 National Electrical and Electronic Experimental Teaching Demonstration Center, School of Electrical and Electronic Engineering, Huazhong University of Science and Technology, Wuhan 430074, China; xuhuiping@hust.edu.cn; 4 Department of Physics, Jiangxi Normal University, Nanchang 330022, China; 5 Department of Physics and Astronomy, Shanghai JiaoTong University, Shanghai 200240, China; zqcao@sjtu.edu.cn

**Keywords:** Goos-Hänchen shift, symmetric metal-cladding waveguide, vortex beam, ultrahigh-order modes

## Abstract

Goos-Hänchen shift provides a way to manipulate the transverse shift of an optical beam with sub-wavelength accuracy. Among various enhancement schemes, millimeter-scale shift at near-infrared range has been realized by a simple symmetrical metal-cladding waveguide structure owing to its unique ultrahigh-order modes. However, the interpretation of the shift depends crucially on its definition. This paper shows that the shift of a Gaussian beam is discrete if we follow the light peak based on the stationary phase approach, where the M-lines are fixed to specific directions and the beam profile is separated near resonance. On the contrary, continuous shift can be obtained if the waveguide is illuminated by a vortex beam, and the physical cause can be attributed to the position-dependent phase-match condition of the ultrahigh-order modes due to the spatial phase distribution.

## 1. Introduction

The Goos-Hänchen shift refers to the lateral displacement of a beam when it is totally or partially reflected at an interface, and has become the subject of various academic and experimental investigations [1,2,3]. The physical origin is simple: a light beam with a finite cross section can be mathematically decomposed into many plane waves with different propagation directions, and the direction-dependent phase changes are combined together to generate the so-called Goos-Hänchen shift. Recently, the Goos-Hänchen effect has been thoroughly investigated for various type of beams, including higher-order Laguerre-Gaussian beams [4], partially coherent light [5], off-axis Airy vortex beams [6], a spin-polarized neutron [7], etc. Since the shift at a single dielectric surface is quite small, i.e., usually subwavelength scale, much attention has been devoted to enhancing this effect by applying different materials and structures. Up to now, the enhancement has been illustrated using gradient metasurfaces [8], epsilon-near-zero slabs [9], weakly absorbing slabs [10], subwavelength gratings [11], graphene [12,13,14], a coherent medium [15], and a cavity optomechanical system [16].

Among various enhancement strategies, a millimeter shift at a near-infrared range has been detected by a position-sensitive detector (PSD) based on a symmetric metal-cladding waveguide (SMCW) structure [17]. The key to a large enhancement is the coupling of the ultrahigh-order modes (UOMs), which can be realized when the intrinsic damping of the structure equals the leakage [18]. First, destructive interference removes the energy propagation along the path predicted by geometric optics; second, the leakage of the propagating guided modes along the guiding layer rebuilds the scattered beam with large lateral shifts. The shift detected by the PSD matches the theoretical result calculated using a stationary phase approach, and both positive and negative lateral shifts can be observed near resonance [19]. The transition between a positive and negative shift is also observed via various other dielectric slabs and structures. However, careful investigation is required before practical application, since the scattered beam usually undergoes severe distortion due to the mode coupling, and the absence of an accurate and reasonable definition of the position of a distorted beam is still a problem. In this paper, we investigate enhancement of the lateral shift of both Gaussian and vortex beams scattered by an SMCW structure. We found that a Gaussian beam splits into two parts as a M-line passes through the beam center at resonance, and the abrupt switch between positive and negative shift is due to the competition between the peak intensity of these two parts. In addition, we have found that the M-line moves continuously across the beam profile and the lateral shift has good tunability if the incident beam is replaced by a vortex beam, meaning that the Goos-Hänchen shift of a vortex beam is not discrete. This result may facilitate practical applications where subtle and adjustable manipulation of light beam with high precision is required. A simple model based on the phase-match condition of the UOMs with a spatial-distributed phase is also proposed to explain the observed phenomenon.

## 2. SMCW Structure and UOMs

**Figure 1:**
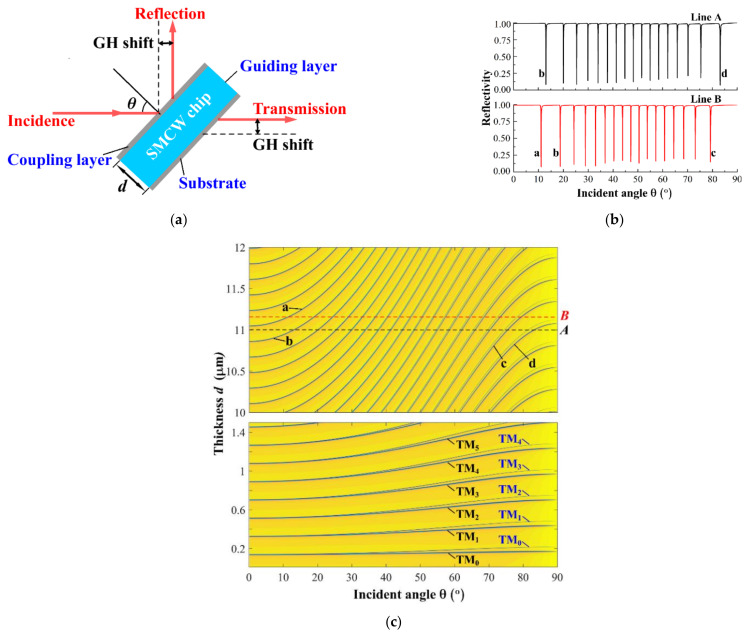
(**a**) The Goos-Hänchen shift of a light wave scattered by the SMCW structure. (**b**) The typical reflection spectra of TM mode of the SMCW with a guiding layer thickness of 11 μm and 11.15 μm, respectively. Other parameters are the same as that used in (**c**), and the reflection curves match the dispersion relations at the dashed lines A and B in (**c**). The resonant dips corresponding to four specific mode lines (a, b, c and d) are also marked out. (**c**) The dispersion relation of the SMCW structure, where the number of the accommodated UOMs increases for thick waveguides.

A SMCW structure is similar to a metal-dielectric-metal (MDM) structure [20], but the former has a guiding layer of millimeter or sub-millimeter thickness [21]. The SMCW structure is simple, and consists of a guiding layer sandwiched between two metallic layers to support the propagation of oscillating modes, i.e., the UOMs. Since it is easy to measure the lateral shift of a transmitted beam that exhibits a fixed propagation direction as the SMCW is subjected to angular scanning, the two metallic layers are 50 nm thick to enable both reflection and transmission. Owing to the free space coupling technology, direct incidence on the waveguide surface is sufficient to excite the UOMs without the application of any couplers. As shown in Figure 1a, the two metallic layers are referred to as the coupling layer and the substrate, respectively. The reflected beam is used to monitor the excitation of the UOMs, and the transmitted beam is applied to measure the lateral shift at different incident angles. The dispersion relation of the SMCW structure is given in Figure 1c, where the curves corresponding to different guided modes are plotted as functions of both the incident angle and the guiding-layer thickness d. The simulated parameters are wavelength λ = 532 nm and the dielectric constant of silver $\varepsilon_{Ag}=-10.6114+0.141i$. It can be seen that a few modes can be excited when the thickness d is small, and the number of accommodated modes increases as the thickness of the guiding layer increases. Further details including the specific reflection spectra for TM polarization corresponding to two horizontal lines A and B are calculated in Figure 1c and displayed in Figure 1b. It is easy to verify that the resonance dips of reflectivity in Figure 1b match perfectly with the crossings of the mode curves and the horizontal lines A and B in Figure 1c. Furthermore, four mode curves are labeled from mode a to d in Figure 1c, and the corresponding resonance dips are also marked out in Figure 1b. Clearly, each mode appears from the left side and disappears to the right side of the reflection spectrum. Consequently, the resonance dip on the left always has a higher mode order. As the mode density increases for thicker waveguides, the full width half maximum of each of the UOMs decreases rapidly and results in high sensitivity to coupling conditions. In addition, it can be seen that the difference between the TE- and TM-polarization states of thicker waveguides decreases rapidly; thus, it proves that the UOMs are polarization-independent.

At this stage, we need to re-examine the definition of the Goos-Hänchen shift based on a stationary-phase approach. Let us consider an integral:

(1) $$I=\int g\left(k\right)e^{i\phi \left(k\right)}dk,$$

where g(k) is a slowly varying function and the phase term ϕ(k) is extremely large. Thus, the exponent term in the integral oscillates rapidly, and these oscillations exhibit a very small net contribution to the total integral. However, if the exponent is stationary, i.e., we have the following equation:

(2) $$\frac{\partial \phi }{\partial k}=0,$$

where we can expect a significant contribution. Similarly, the Goos-Hänchen shift predicted by the stationary-phase approach is:

(3) $$S=-\frac{1}{k}\frac{d\phi }{d\theta },$$

where θ is the incident angle. According to the above discussion, the beam position is defined by the position of its intensity peak. Problems would arise in relation to the validity of this definition if the beam undergoes significant distortion, but this issue is beyond the scope of this paper.

## 3. Enhanced Lateral Shift for Gaussian and Vortex Beam

**Figure 2:**
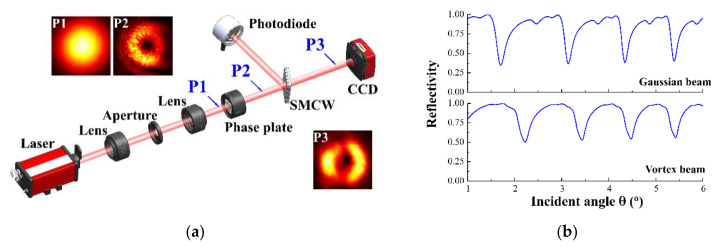
(**a**) Experimental setup of the enhancement and detection of the Goos-Hänchen shift effect via the scattering of a vortex beam on an SMCW structure. Typical light patterns at three different positions (P1, P2 and P3) in the light path are displayed in the insets. (**b**) Comparison of the typical reflection spectra measured by the photodetector between incidences of Gaussian beam and vortex beam.

Liu et al. found a large positive and negative lateral optical beam shift in a prism-waveguide coupling system [22]. In this paper, we use an SMCW structure via a free-space coupling to enhance the Goos-Hänchen shift. Compared with a Gaussian beam, a vortex beam has a spiral phase and energy that circles around the central phase singularity [23]. A schematic diagram of Goos-Hänchen shift detection via scattering on an SMCW structure is plotted in Figure 2a. A 532 nm laser beam passes through a 4f system consisting of two lenses and an aperture to enable further collimation. A phase plate with a topological charge of l=1 is inserted into the light path to generate an optical vortex. The SMCW structure used in our experiment was produced by coating a silver film on both sides of a 0.15 mm thick glass slab that exhibits excellent optical parallelism. Angular scanning of the SMCW structure was carried out by a computer-controlled θ/2θ goniometer, in which a photodetector rotates at a double angular speed around the SMCW to record reflectivity. As a specific UOM was excited, energy transferred from the reflected beam into the guiding layer and thereby enabled a resonance dip that was observed in the reflection spectrum. The direction of the transmitted beam is invariant under rotation of the SMCW, and a charge-coupled device (CCD) was applied to monitor lateral shift and distortion. Experimental images of the beam at three different positions in the light path are shown as insets in Figure 2, including the Gaussian beam (P1), the vortex beam (P2) and the transmitted vortex beam (P3). In Figure 2b, the typical reflectivity of a Gaussian beam and a vortex beam are compared, in which the coupling depth of the Gaussian beam is larger than the vortex beam. This can be explained as follows. The wave vector for a Gaussian beam is distributed tightly around the central beam vector, so most energy can be coupled into the guided mode as the incident angle reaches a phase-match condition. On the other hand, the wave front of a vortex beam has a spiral spatial structure, so its wave vector has a broad distribution, and the coupling efficiency is lower [24,25].

**Figure 3:**
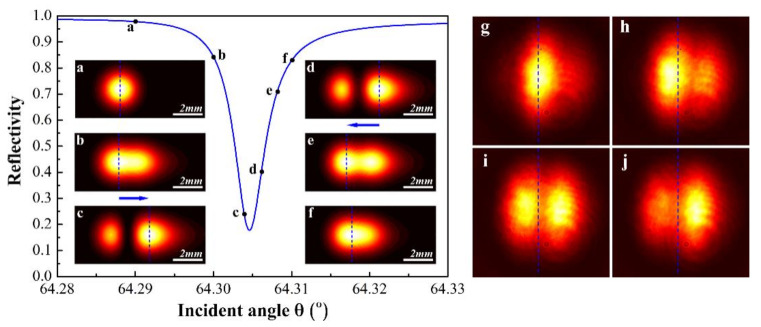
The discrete Goos-Hänchen shift effect of a Gaussian beam due to the UOMs coupling. (**a**–**f**) Simulated light patterns within a single resonant dip, where a M-line divided the light profile into two separated parts near resonance. The blue lines mark the position of the intensity peaks. (**g**–**j**) Experimentally measured results confirm the discrete negative and positive shift during a specific UOM coupling, where the blue dashed lines mark the position of the initial beam center in (**g**) for better comparison.

A Fourier-transformation-based beam propagation method was applied to simulate the transmitted light pattern of the Gaussian beam during excitation of a specific UOM. In Figure 3, the resonance dip around an incident angle of 64° was calculated by the transfer matrix method [26], and the light pattern examples at six different incident angles are simulated and displayed in Figure 3a–f, whilst the corresponding incident angles are marked out as black dots on the reflection curve. Figure 3a shows that the reflectivity is close to unit and the UOM is not coupled. Therefore, most energy is reflected, and the beam pattern is not distorted. Figure 3b illustrates beam stretching as the incident angle approaches the resonant angle. In Figure 3c,d, an M-line appears at the beam center and its position is fixed. If the beam position is defined with reference to its intensity peak, an abrupt positive shift can be predicted since the right peak intensity is higher than the left peak intensity. In Figure 3e, the fixed M-line gradually disappears resulting in the merger of the separated two parts as the incident angle passes the resonant angle. An abrupt negative shift can be predicted as the left peak intensity exceeds the right peak intensity. Note that there are still two peaks in Figure 3e, so the lateral shift is not continuous since neither peak moves continuously across the beam cross-section. For example, in Figure 3f, as the incident angle is further away from the resonant angle, the shape of the beam gradually returns to a Gaussian beam.

Figure 3g–j show the experimentally-measured distortions of the Gaussian beam during a UOM excitation, which fit well with the theoretical simulations. Figure 3g shows the original transmitted beam. Figure 3h illustrates appearance of the M-line and observation of a negative shift. In Figure 3i, the beam splits into two parts, and the right peak intensity increases. In Figure 3j, an abrupt change from a negative shift to a large positive shift is observed when the right part has a higher peak intensity. The above experimental observations confirm that an M-line corresponding to the UOM coupling does not move in space, it appears in a specific direction and it divides the light spot into two parts. If the lateral shift is defined by the intensity peak of the beam, both positive and negative shifts may occur, and the transition between the positive and negative shifts is discrete.

**Figure 4:**
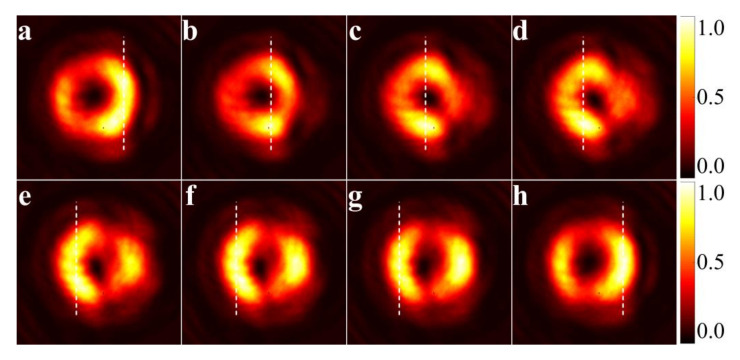
Experimentally measured Goos-Hänchen shift of a vortex beam with unit topological charge. The white dashed lines mark out the positions of the pattern peak, which shift continuously from left to right as the incident angle increases. Each figure is normalized to its maximal value to illustrate further detail.

The lateral shift for the vortex beam is quite different, and Figure 4 shows the evolution of the light pattern of a transmitted vortex beam during the excitation of a UOM. As the incident angle sweeps over each resonant dip, the intensity peak moves continuously from the left side to the right side of the beam. Most importantly, there is no fixed M-line to act as a forbidden region for lateral shift. If we follow the stationary-phase approach and define the Goos-Hänchen shift with reference to the location of the position of the intensity peak, vortex-beam incidence can result in a continuous lateral shift. In addition, continuous displacement exhibits good tunability in response to changing the incident angle. A simple explanation is proposed as follows.

**Figure 5:**
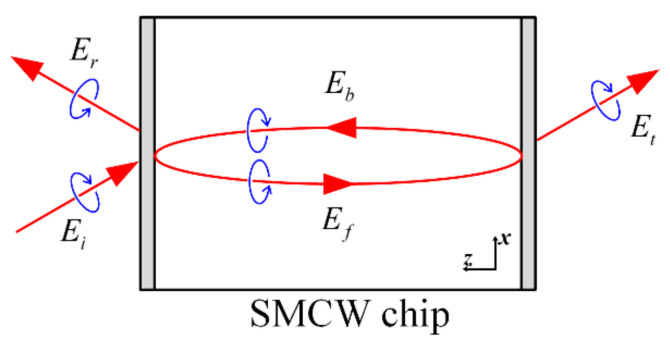
A simple model of the scattering of the vortex beam carrying angular momentum on an SMCW structure.

In Figure 5, the incidence, reflection and transmission are labeled as $E_{i},\;E_{r}$ and $E_{t}$, respectively. The SMCW structure is simplified as a Fabry-Perot cavity, and the mode inside the guiding layer is decomposed into a forward component $E_{f}$ and a backward component $E_{b}$. Above all, these two components carry opposite topological charge, thus the phase-match condition for the vortex beam can be approximated as

(4) $$kd+\cos^{-1}\frac{x}{\sqrt{x^{2}+y^{2}}}=m\pi ,\;\;\;\;\;m=1,2,3,\ldots ,$$

where $k=\frac{2\pi }{\lambda }{\left(\varepsilon_{g}-\varepsilon_{0}\sin^{2}\theta \right)}^{1/2}$ is the longitude wavenumber in the guiding layer with dielectric constant $\varepsilon_{g}$ and thickness d, and m is the mode order. In comparison with the ordinary phase-match condition [27],

(5) kd=mπ,     m=1,2,3,…,

and an additional phase term is added to Equation (4), so the phase-match condition becomes coordinate-dependent. Therefore, for the Gaussian beam, the phase-match condition is the same for the whole cross-section, and the M-line is fixed roughly in the beam center. This is not the case for a vortex beam, the coordinate-dependent excitation of the UOM leads to the continuously tunable lateral shift of the peak position. In addition, the proposed hypothesis matches experimental observation.

**Figure 6:**
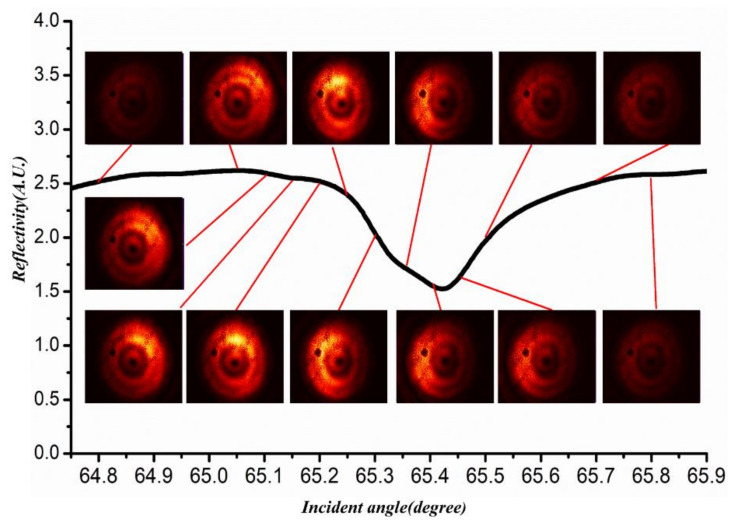
Goos-Hänchen shift of a transmitted vortex beam with topological charge l=1 in one resonance period. The succession of beam profiles is normalized to the highest intensity during the process of resonance for a better view of the evolution of the transmitted beam.

The Figure 6 shows the reflectivity at different incident angles with transmitted patterns, i.e., the occurrence of Goos-Hänchen shift effect, within a single resonance period. The maximum of vortex beam intensity moves continuously with the variation of the incident angle, resulting in the continuous positive and negative shift. Moreover, the intensity of the central ring is not the strongest due to the inter-mode coupling effect of the waveguide and the vortex beam with topological charge of one exhibits the Goos-Hänchen shift effect only once in a guided mode resonance period.

## 4. Conclusions

In this paper, a continuous and tunable Goos-Hänchen shift is observed when the SMCW structure is excited by a vortex beam. We prove that the lateral shift of a transmitted Gaussian beam is discrete, and an abrupt variation between positive shift and negative shift may occur if a stationary phase approach is applied. In contrast, the shift enhancement of a vortex beam is due to the excitation of the UOMs, whilst the continuous shift effect can be attributed to the coordinate-dependent phase-match condition of the UOMs.
